# Synergistic Effects of *Pyrrosia lingua* Caffeoylquinic Acid Compounds with Levofloxacin Against Uropathogenic *Escherichia coli*: Insights from Molecular Dynamics Simulations, Antibiofilm, and Antimicrobial Assessments

**DOI:** 10.3390/molecules29235679

**Published:** 2024-11-30

**Authors:** Yan Zhang, Fangfang Jiao, Derong Zeng, Xiang Yu, Yongqiang Zhou, Juan Xue, Wude Yang, Jingjing Guo

**Affiliations:** 1 College of Pharmacy, Guizhou University of Traditional Chinese Medicine, Guiyang 550025, China; zhangyan0003@gzy.edu.cn (Y.Z.); zengderogs@foxmail.com (D.Z.); yuxiang@gzy.edu.cn (X.Y.); zhouxiaoqiang1988@126.com (Y.Z.); xuejuan062@gzy.edu.cn (J.X.); 2 Centre in Artificial Intelligence Driven Drug Discovery, Faculty of Applied Sciences, Macao Polytechnic University, Macao, China; p2212238@mpu.edu.mo

**Keywords:** UTIs, UPEC, *Pyrrosia lingua*, levofloxacin, molecular simulation

## Abstract

Urinary tract infections (UTIs), primarily caused by uropathogenic *Escherichia coli* (UPEC), have high morbidity and recurrence rates. Resistance to levofloxacin hydrochloride (LEV), a commonly used treatment for UTIs, is increasingly problematic, exacerbated by biofilm formation mediated by interactions between cyclic di-GMP (c-di-GMP or CDG) and YcgR. In this study, we identified three caffeoylquinic acid compounds from *Pyrrosia lingua*—chlorogenic acid (CGA), sibiricose A5 (Si-A5), and 3-*O*-caffeoylquinic acid methyl ester (CAM)—that target YcgR through molecular docking. Biological assays revealed that combining these compounds with levofloxacin hydrochloride significantly enhanced antibacterial activity against standard UPEC strains in a concentration-dependent manner and clinically isolated UPEC strains. Notably, chlorogenic acid and sibiricose A5, when used with levofloxacin hydrochloride, enhanced intracellular c-di-GMP levels and swimming motility, significantly reduced YcgR gene expression, and effectively inhibited biofilm formation of UPEC at multiple time points. Additionally, molecular dynamics simulations elucidated the strong binding of these compounds to YcgR, underscoring the critical roles of residues, such as Arg118 and Asp145. This research serves as a foundation for tackling antibiotic resistance and developing innovative therapeutics for UTIs.

## 1. Introduction

Urinary tract infections (UTIs) are the second most common infectious disease, primarily caused by uropathogenic *Escherichia coli* (UPEC). UPEC accounts for 70–95% of UTIs [1,2]. Globally, approximately 150 million people suffer from UTIs each year, and at least one-third of them will develop recurrent infections [3]. The high morbidity and recurrence rates lead to great burdens on society and public health [4]. Currently, antibiotics are mainly used to treat UTIs; however, antibiotic resistance has been a global problem. Biofilms serve as reservoirs of infection, making it difficult to kill bacteria even at concentrations far beyond the minimum inhibitory concentration (MIC) [5]. When UPEC forms biofilms, it becomes much more difficult to eradicate with antibiotics, leading to recurring UTIs that are challenging to treat effectively [6].

Cyclic diguanosine monophosphate (c-di-GMP or CDG), serving as a ubiquitous second messenger across bacterial species, plays a key role in regulating biofilm dispersal and formation. An elevated cellular concentration of c-di-GMP binds the C terminal conserved PilZ domain of the only flagellar brake protein YcgR of UPEC. This interaction allows the N-terminal domain of YcgR (YcgR-N) to stably interact with motor proteins MotA, thereby inhibiting the MotA-FliG association and reducing bacterial motility. As a result, it increases matrix component production and promotes biofilm formation. However, a low cellular concentration of c-di-GMP promotes biofilm dispersal through increased bacterial motility. The YcgR mutant with an Arg118Asp substitution cannot associate with c-di-GMP and, therefore, cannot interact with the FliG protein. The mechanism by which an elevated cellular concentration of c-di-GMP induces a structural rearrangement of the PilZ and YcgR-N domains of YcgR is not clear, yet it is vital for biofilm formation and dispersal [7,8,9,10]. This understanding is crucial, because the use of antibiotics alone can easily lead to the development of antimicrobial drug resistance.

Natural products and synthetic compounds alone and combined with antibiotics including ciprofloxacin, such as citrus peel extract, cinnamaldehyde, resveratrol, disperazol, and zingerone, inhibit bacterial biofilm formation by regulating c-di-GMP concentrations and enhancing antibiotic efficacy [11,12,13,14,15]. *Pyrrosia lingua* has diuretic and strangury-relieving properties and has been widely used to treat UTIs in combination with antibiotics. The herbal remedy has advantages in terms of low side effects, low risk of drug resistance, and high efficacy [16,17,18]. According to previous studies, *Pyrrosia lingua* contains high levels of caffeoylquinic acid compounds, such as chlorogenic acid (CGA), sibiricose A5 (Si-A5), and 3-*O*-caffeoylquinic acid methyl ester (CAM) [19,20]. These compounds have anti-inflammatory, antimicrobial, antioxidant, antitumor, hypolipidemic, and hypoglycemic activities [21,22,23]. Despite the potential benefits, there are limited reports on the use of chlorogenic acid analogs in combination with antibiotics to enhance their antibacterial and anti-biofilm activities regulating c-di-GMP [24,25].

Our molecular docking studies indicate that these three compounds bind well with YcgR [20,26,27]. Given the promising results from our docking studies and the known properties of chlorogenic acid, sibiricose A5, and 3-*O*-caffeoylquinic acid methyl ester, we aimed to further investigate their combined effects with antibiotics against UPEC. We employed methods such as disk diffusion, broth microdilution, microdilution checkerboard, crystal violet assays, swimming motility assay, enzyme-linked immunosorbent assay (ELISA) and quantitative real-time PCR (qRT-PCR) to evaluate their synergistic antibacterial and anti-biofilm activities. Additionally, we explored the regulatory mechanism of these compounds on the interaction between YcgR and c-di-GMP through molecular dynamics simulations.

Experimental results suggest that they exhibit additive or synergistic effects when used in combination with antibiotics. This combination approach can help mitigate the issues associated with the use of antibiotics alone, such as the development of antimicrobial drug resistance. Our findings based on simulations suggest that these compounds may regulate the interaction between YcgR and c-di-GMP, potentially reducing antibiotic resistance and preventing recurrent UTIs. This underscores the potential of combining herbal remedies with conventional antibiotics to enhance treatment efficacy and combat drug-resistant infections. Additionally, understanding the synergistic mechanisms of caffeoylquinic acid compounds with antibiotics is crucial for reducing UTI recurrence and antibiotic resistance, providing significant insights and guiding value for future treatments.

## 2. Results and Discussion

### 2.1. Enhanced Antibacterial Activity of Caffeoylquinic Acid Compounds Combined with Levofloxacin Hydrochloride at Various Concentrations

**Figure 1:**
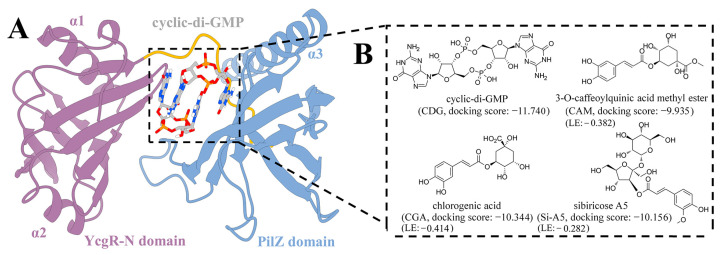
System information. (**A**) A cartoon representation of the YcgR-CDG complex structure. The YcgR-N domain is colored purple, and the PilZ domain is colored blue. The two c-di-GMP molecules are shown as sticks, with carbon atoms in gray, oxygen atoms in red, nitrogen atoms in blue, and phosphorus atoms in orange. The binding site of c-di-GMP is highlighted with a dashed box. (**B**) Chemical structures of c-di-GMP and three caffeoylquinic acid compounds studied in this work with their docking scores (kcal/mol) and ligand efficiency (LE, kcal/(mol·heavy atom)).

To identify potential antibacterial compounds, molecular docking studies were performed on the compounds previously isolated from *Pyrrosia lingua* by our research group. As a result, three caffeoylquinic acid compounds exhibited strong binding with the active site of YcgR, such as chlorogenic acid, sibiricose A5, and 3-*O*-caffeoylquinic acid methyl ester, the docking scores were −10.344, −10.156, and −9.935 kcal/mol, and the corresponding ligand efficiency (LE) were −0.282, −0.382, and −0.414 kcal/(mol·heavy atom), respectively (Figure 1). These three compounds were selected for further evaluation of their antibacterial activity independently and in combination with levofloxacin hydrochloride (LEV).

As detailed in Table 1, the antibacterial efficacy of chlorogenic acid, sibiricose A5, and 3-*O*-caffeoylquinic acid methyl ester, both as standalone agents and in combination with LEV, was assessed against UPEC using the disk diffusion method at concentrations of 25, 50, 100, 200, and 400 μg/mL, respectively. Levofloxacin hydrochloride alone inhibited *E. coli* ATCC 10389 with zone diameters ranging from 15.5 to 27.7 mm. When combined with the caffeoylquinic acid compounds, the inhibition zones increased, measuring between 16.4 and 30.6 mm. Additionally, the antibacterial activity of levofloxacin hydrochloride and its combinations improved as the concentration increased.

As shown in Table 1, when administered alongside levofloxacin hydrochloride, chlorogenic acid, sibiricose A5, and 3-*O*-caffeoylquinic acid methyl ester achieved inhibition zone diameters (IZDs) of 16.4 mm, 17.9 mm, and 19.2 mm, respectively. For comparison, levofloxacin hydrochloride alone produced an IZD of 16.3 mm. Notably, all combinations exhibited superior antibacterial activity compared to levofloxacin hydrochloride alone, with 3-*O*-caffeoylquinic acid methyl ester showing the most significant enhancement of the synergistic antibacterial effects.

### 2.2. Synergetic Antibacterial and Antibiofilm Activities of Caffeoylquinic Acid Compounds and Levofloxacin Hydrochloride

The results of the synergistic antibacterial activities of caffeoylquinic acid compounds with levofloxacin hydrochloride, assessed using the broth microdilution method, are presented in Table 2. These compounds exhibited synergistic effects with levofloxacin hydrochloride against the standard strain ATCC 10389 and clinically isolated UPEC 64222 and 55758 strains, with the fractional inhibitory concentration index (FIC) ranging from 0.25 to 0.75. Specifically, the minimum inhibitory concentrations (MICs) of the antibiotic had a 2–8-fold reduction when used in combination. Chlorogenic acid achieved a 4–16-fold reduction, while both sibiricose A5 and 3-*O*-caffeoylquinic acid methyl ester demonstrated an 8-fold and 4–16-fold reduction in MIC, respectively. Moreover, compared with clinically isolated UPEC strains, caffeoylquinic acid compounds and levofloxacin hydrochloride in single and combined use showed better antimicrobial efficacy against the standard strain. This synergy not only enhanced the antimicrobial efficacy but also reduced the likelihood of resistance development, addressing a critical clinical healthcare challenge.

**Figure 2:**
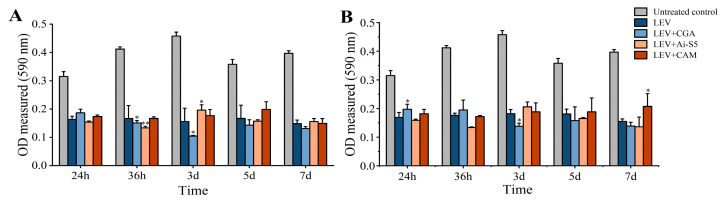
OD values of levofloxacin hydrochloride in combination with caffeoylquinic acid compounds against *E. coli* ATCC 10389 at multiple time points and concentrations. (**A**) OD values of levofloxacin hydrochloride and caffeoylquinic acid compounds at MIC concentrations; (**B**) OD values of levofloxacin hydrochloride and caffeoylquinic acid compounds at 1/2 MIC concentrations. The single (*) and double (**) asterisks represent *p* < 0.05 and *p* < 0.01, compared with levofloxacin hydrochloride, respectively.

Based on the better antimicrobial efficacy of caffeoylquinic acid compounds and levofloxacin hydrochloride in single and combined use, the effects of the antibiotic and these compounds on biofilm formation across the standard strain were analyzed. Levofloxacin hydrochloride significantly inhibited biofilm formation at multiple time points, including 24 h, 36 h, 3 days, 5 days, and 7 days (Figure 2). Additionally, the combinations of chlorogenic acid or sibiricose A5 with levofloxacin hydrochloride markedly reduced biofilm formation across the standard strain at different time points. In contrast, 3-*O*-caffeoylquinic acid methyl ester did not inhibit biofilm formation. While increasing the concentration of levofloxacin hydrochloride slightly enhanced its ability to inhibit biofilm formation, decreasing the concentration of 3-*O*-caffeoylquinic acid methyl ester was beneficial for biofilm inhibition. Furthermore, higher concentrations of chlorogenic acid were particularly effective in reducing biofilm formation.

### 2.3. Swimming Motility Inhibition Activities of Caffeoylquinic Acid Compounds and Levofloxacin Hydrochloride

**Figure 3:**
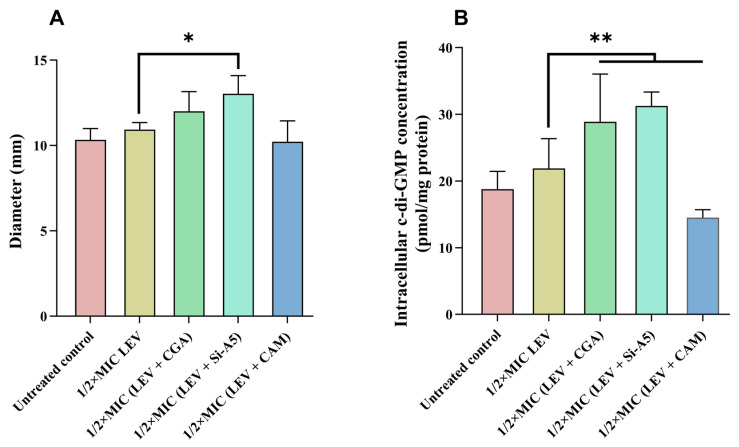
Effects of levofloxacin hydrochloride and caffeoylquinic acid compounds at 1/2 MIC concentrations on swimming motility and intracellular c-di-GMP concentrations in *E. coli* ATCC 10389. (**A**) Swimming motility inhibition activities of *E. coli* ATCC 10389 treated with levofloxacin hydrochloride and caffeoylquinic acid compounds at 1/2 MIC concentrations; (**B**) Intracellular c-di-GMP concentrations in *E. coli* ATCC 10389 treated with levofloxacin hydrochloride and caffeoylquinic acid compounds at 1/2 MIC concentrations. The data are expressed as the mean ± SD of at least three independent experiments. The single (*) and double (**) asterisks represent *p* < 0.05 and *p* < 0.01, compared with 1/2 MIC concentration of levofloxacin hydrochloride, respectively.

As illustrated in Figure 3A, the swimming motility of *E. coli* ATCC 10389, when treated with levofloxacin hydrochloride at 1/2 MIC concentration, did not significantly increase compared with the untreated control group. However, the combinations of levofloxacin hydrochloride with chlorogenic acid or sibiricose A5 resulted in notable enhancements in swimming ability, especially sibiricose A5 (*p* < 0.05). In contrast, levofloxacin hydrochloride combined with 3-*O*-caffeoylquinic acid methyl ester led to a reduction in swimming ability. Consistently, the combinations of chlorogenic acid or sibiricose A5 with levofloxacin hydrochloride markedly reduced biofilm formation, while its combination with 3-*O*-caffeoylquinic acid methyl ester did not inhibit biofilm formation. These findings suggested that levofloxacin hydrochloride combined with chlorogenic acid or sibiricose A5 enhanced the swimming motility of *E. coli* ATCC 10389 and reduced biofilm formation, whereas its combination with 3-*O*-caffeoylquinic acid methyl ester reduced the swimming motility of *E. coli* ATCC 10389 and did not inhibit biofilm formation.

### 2.4. Intracellular c-di-GMP Concentrations Regulated by Caffeoylquinic Acid Compounds and Levofloxacin Hydrochloride

To study the correlation between biofilm formation, swimming motility, and intracellular c-di-GMP levels of *E. coli* ATCC 10389, intracellular c-di-GMP concentrations in *E. coli* ATCC 10389 were measured when treated with levofloxacin hydrochloride and caffeoylquinic acid compounds at 1/2 MIC concentrations. As shown in Figure 3B, levofloxacin hydrochloride alone did not significantly affect the intracellular c-di-GMP content of *E. coli* ATCC 10389. However, the c-di-GMP content of *E. coli* ATCC 10389 was significantly increased after levofloxacin hydrochloride in combination with chlorogenic acid or sibiricose A5 (*p* < 0.01). In contrast, c-di-GMP content was decreased after levofloxacin hydrochloride in combination with 3-*O*-caffeoylquinic acid methyl ester (*p* < 0.01). These results suggested that levofloxacin hydrochloride in combination with chlorogenic acid or sibiricose A5 increased intracellular c-di-GMP content of *E. coli* ATCC 10389. However, the combination of levofloxacin hydrochloride and 3-*O*-caffeoylquinic acid methyl ester decreased c-di-GMP content. Thus, levofloxacin hydrochloride, in combination with chlorogenic acid or sibiricose A5, enhanced intracellular c-di-GMP levels, swimming motility, and reduced biofilm formation. In contrast, the combination of CAM and LEV resulted in similar swimming motility patterns as the control and the LEV-only treatments (Figure 3A), and the intracellular c-di-GMP concentration in the CAM + LEV system remains even lower than the levofloxacin-only and control groups.

### 2.5. Significant Reduction in YcgR Flexibility Due to Ligand Binding

Next, molecular dynamics (MD) simulations were performed to elucidate the binding interactions between the YcgR protein and CDG (PDB ID: 5Y6F [10]), as well as the three caffeoylquinic acid compounds. Here, we established seven systems: YcgR-apo, representing the unbound state of the YcgR protein; YcgR-CDG-dual, featuring YcgR complexed with two c-di-GMP molecules; YcgR-CDG-PilZ, showing YcgR with a c-di-GMP molecule close to the PilZ domain; and YcgR-CDG-N domain, with a c-di-GMP molecule close to the YcgR-N domain. In addition, YcgR-CDG-dual-PilZ refers to the complex formed by a c-di-GMP molecule located near the PilZ domain within the YcgR-CDG-dual complex. Conversely, the YcgR-CDG-dual-N domain denotes the complex formed by a c-di-GMP molecule situated near the N-domain within the YcgR-CDG-dual complex. Moreover, YcgR-CAM refers to the complex of YcgR bound with 3-*O*-caffeoylquinic acid methyl ester, YcgR-CGA represents the complex of YcgR with chlorogenic acid, and YcgR-Si-A5 stands for YcgR bound to sibiricose A5. 

**Figure 4:**
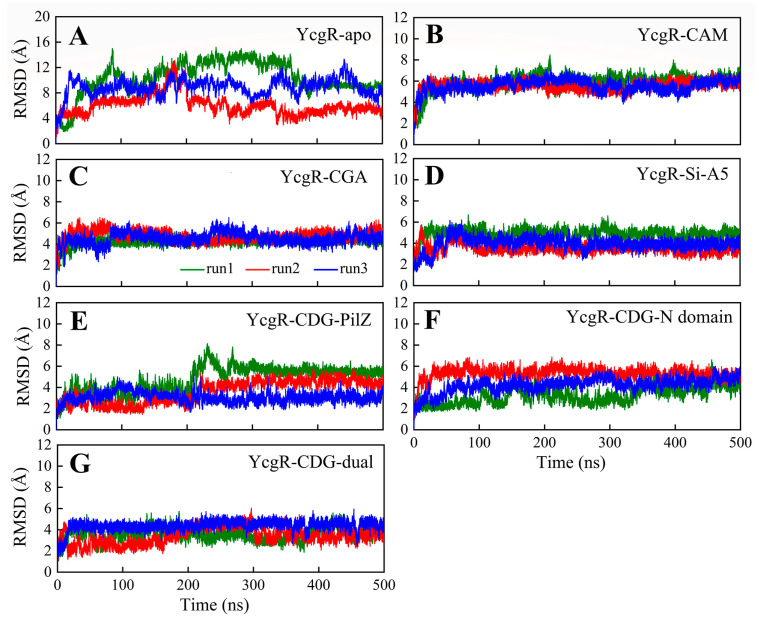
The RMSDs of all simulated systems during the MD simulations: (**A**) YcgR-apo system; (**B**) YcgR-CAM system; (**C**) YcgR-CGA system; (**D**) YcgR-Si-A5 system; (**E**) YcgR-CDG-PilZ system; (**F**) YcgR-CDG-N domain system; (**G**) YcgR-CDG-dual system. Each system is carried out in triplicate.

The root-mean-square deviations (RMSDs) of the Cα atoms of YcgR were calculated by aligning to the initial conformation to evaluate the structural stability of each simulated system. As illustrated in Figure 4, the RMSD values of these seven systems were stable after the 500 ns MD simulations, but the RMSD value (<12 Å) for the YcgR-apo system was larger than the other six YcgR-ligand complexes (<6 Å). In addition, YcgR-CDG-dual, YcgR-CDG-PilZ, and YcgR-Si-A5 exhibited the lowest RMSD values (~4 Å) among all simulated systems, which were attributed to their larger molecular skeletons acting as a bridge and stabilizing the conformation between the PilZ and YcgR-N domains (Figure 1A). Moreover, caffeoylquinic acid compounds significantly reduced the flexibility of PilZ and YcgR-N domains, especially chlorogenic acid and sibiricose A5. Hence, the presence of ligands between the PilZ domain and the YcgR-N domain should be beneficial to the stability of YcgR.

### 2.6. The Binding Mechanism of YcgR and Caffeoylquinic Acid Compounds

To further evaluate the binding mechanism between YcgR and ligands, the MM-GBSA method was employed to calculate the binding free energy for the YcgR-ligand complexes over the last 50 ns MD trajectories. Entropic contribution (TΔS) was not considered here due to the high resource consumption. As listed in Table 3 and Table 4, the average binding free energies (Δ*G*_MM/GBSA_) were −29.59, −31.87, −42.48, −44.12, −33.30, −55.89, and −40.52 kcal/mol for complexes YcgR-CAM, YcgR-CGA, YcgR-Si-A5, YcgR-CDG-PilZ, YcgR-CDG-N domain, YcgR-CDG-dual-PilZ (a c-di-GMP molecule near the PilZ domain in YcgR-CDG-dual), and YcgR-CDG-dual-N domain (a c-di-GMP molecule near the N-domain in YcgR-CDG-dual), respectively. Two c-di-GMP were more favorable for the association between c-di-GMP and YcgR than one c-di-GMP. Moreover, the c-di-GMP adjacent to the PilZ domain was more favorable. Caffeoylquinic acid compounds exhibited strong binding with YcgR, especially chlorogenic acid and sibiricose A5. In detail, the van der Waals interaction (Δ*E*_vdw_) and the direct electrostatic interaction (Δ*E*_ele_) were the crucial driving forces for the binding between ligands and YcgR. In contrast, the polar component (Δ*G*_pol,sol_) was not conducive to the ligand binding. It is noteworthy that chlorogenic acid and sibiricose A5, which bound well with YcgR, were unfavorable for the association between c-di-GMP and YcgR. This led to enhanced intracellular c-di-GMP levels, swimming motility, and reduced biofilm formation. However, 3-*O*-caffeoylquinic acid methyl ester bound weakly with YcgR had weak effects on the association between YcgR and c-di-GMP, which led to reduced c-di-GMP levels and swimming motility. It is well known that levofloxacin hydrochloride targets DNA gyrase and thus has no obvious effects on c-di-GMP levels and swimming motility. CAM may exert its synergistic antimicrobial effects through alternative mechanisms, rather than the c-di-GMP-mediated motility regulation observed with CGA and Si-A5.

**Figure 5:**
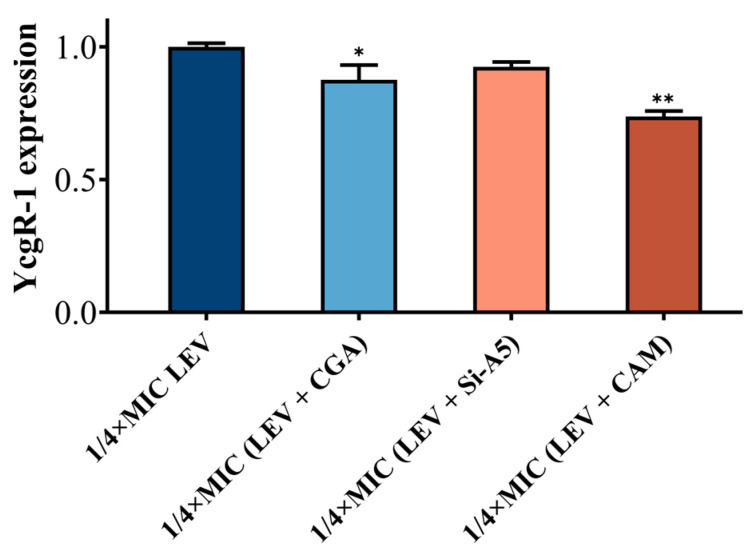
Effects of levofloxacin hydrochloride and caffeoylquinic acid compounds on YcgR gene expression in *E. coli* ATCC 10389 at 1/4 MIC concentration. The single (*) and double (**) asterisks represented *p* < 0.05 and *p* < 0.01, compared with 1/4 MIC concentration of levofloxacin hydrochloride, respectively.

Owing to the effects of the association of caffeoylquinic acid compounds and YcgR on c-di-GMP levels, swimming motility, and biofilm formation, the relative expression of YcgR in *E. coli* ATCC 10389 was examined with either levofloxacin hydrochloride alone or levofloxacin hydrochloride combined with caffeoylquinic acid compounds at a concentration of 1/4 MIC for 4 h. As shown in Figure 5, levofloxacin hydrochloride combined with caffeoylquinic acid compounds significantly reduced YcgR gene expression in UPEC. Specifically, the LEV + CGA and LEV + Si-A5 groups resulted in 1.14-fold and 1.08-fold reductions in YcgR expression, respectively. In contrast, the LEV + CAM group exhibited a 1.36-fold reduction in YcgR expression. Consistently, CGA and Si-A5 with YcgR had more stable binding while CAM with YcgR had weak association. Therefore, the combination of levofloxacin hydrochloride and caffeoylquinic acid compounds exerted an inhibitory effect on the expression of the YcgR-1 gene through the binding of caffeoylquinic acid compounds and YcgR.

The identified caffeoylquinic acid compounds significantly inhibited the interaction between c-di-GMP and YcgR, disrupting biofilm formation pathways that are key in antibiotic resistance and bacterial persistence. These findings offer a deeper understanding of how these compounds could be applied to resolve biofilm-associated infections. In addition, as natural products obtained from *Pyrrosia lingua*, these compounds have advantages such as low toxicity and compatibility with existing antibiotics. They strengthened the contributions of traditional herbal medicine to modern therapeutic solutions.

### 2.7. The Hotspot Residues in YcgR-Ligand Binding

**Figure 6:**
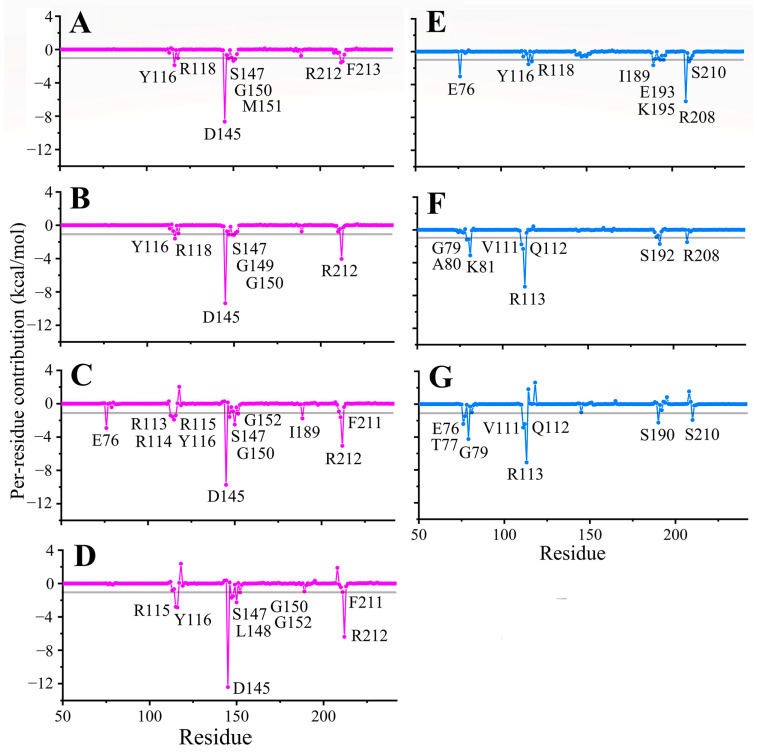
Residue contribution to the binding of ligands: (**A**) YcgR-CAM system; (**B**) YcgR-CGA system; (**C**) YcgR-CDG-PilZ system; (**D**) YcgR-CDG-dual-PilZ system; (**E**) YcgR-Si-A5 system; (**F**) YcgR-CDG-N domain system; (**G**) YcgR-CDG-dual-N domain system.

The binding free energies were decomposed at the per-residue level to identify the critical residues involved in YcgR-ligand interactions. Residues with a per-residue contribution lower than −1 kcal/mol were considered hotspot residues. As shown in Figure 6, Figure S1 and S2, two different interaction patterns were identified in all simulated systems. In detail, YcgR-CAM, YcgR-CGA, YcgR-CDG-PilZ, and YcgR-CDG-dual-PilZ mainly bound to the upper region of the PilZ domain (Figure S3), and residue Y116, D145, S147, G150, and R212 were identified as the common hotspot residues in these four complexes, especially residue D145 exhibited high interaction energy among all the key residues. In addition, YcgR-Si-A5, YcgR-CDG-N domain, and YcgR-CDG-dual-N domain bound to the lower region of the PilZ domain and YcgR-N domain (Figure S4), and residue R208 or R113 was identified as one of the common hotspot residues in these three systems. Additionally, hotspot residues in these three systems changed significantly. For example, R118 was favorable for the binding of caffeoylquinic acid compounds. However, it was unfavorable for the binding of the two c-di-GMP molecules and showed no significant contribution to the one c-di-GMP system.

### 2.8. The Binding Mechanisms Between YcgR and Ligands

**Figure 7:**
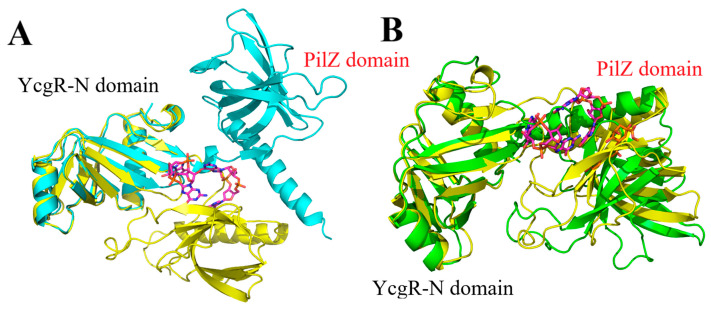
A cartoon representation of YcgR-CDG-dual (yellow) and apo YcgR (cyan). C-di-GMP was shown in pink (**A**). A cartoon representation of YcgR-CDG-dual (yellow) and YcgR-CAM (green) (**B**). C-di-GMP and 3-*O*-caffeoylquinic acid methyl ester were shown in stick representation, with carbon atoms colored pink (C-di-GMP) or orange (3-caffeoylquinic acid methyl ester), oxygen atoms in red, nitrogen atoms in blue, and phosphorus atoms in orange.

Based on the further structural analysis of the YcgR-CDG-dual and apo YcgR, the V-shape conformation of apo YcgR derived from the experimental structure of CDG-YcgR complex became linear during MD simulation, with the two YcgR subdomains moving to their farthest separation (Figure 7). However, c-di-GMP binding led to the PilZ domain being closer to the YcgR-N domain. As a result, YcgR exhibited a compact V-shape conformation, and the structure of YcgR-N domain was almost invariant. Similarly, YcgR-CDG-dual and YcgR-CAM had compact V-shape structures, and the PilZ and YcgR-N domains of YcgR-CDG-dual were nearer. Consistent with previous studies [7,8,9,10], an elevated cellular concentration of c-di-GMP binds the PilZ domain and reduces bacterial motility through its interaction with the YcgR-N domain, thereby facilitating biofilm formation. The ligand primarily binds to the PilZ domain, bringing the PilZ and YcgR-N domains into close proximity. Thus, the flexibility of YcgR and bacterial motility will be reduced.

**Figure 8:**
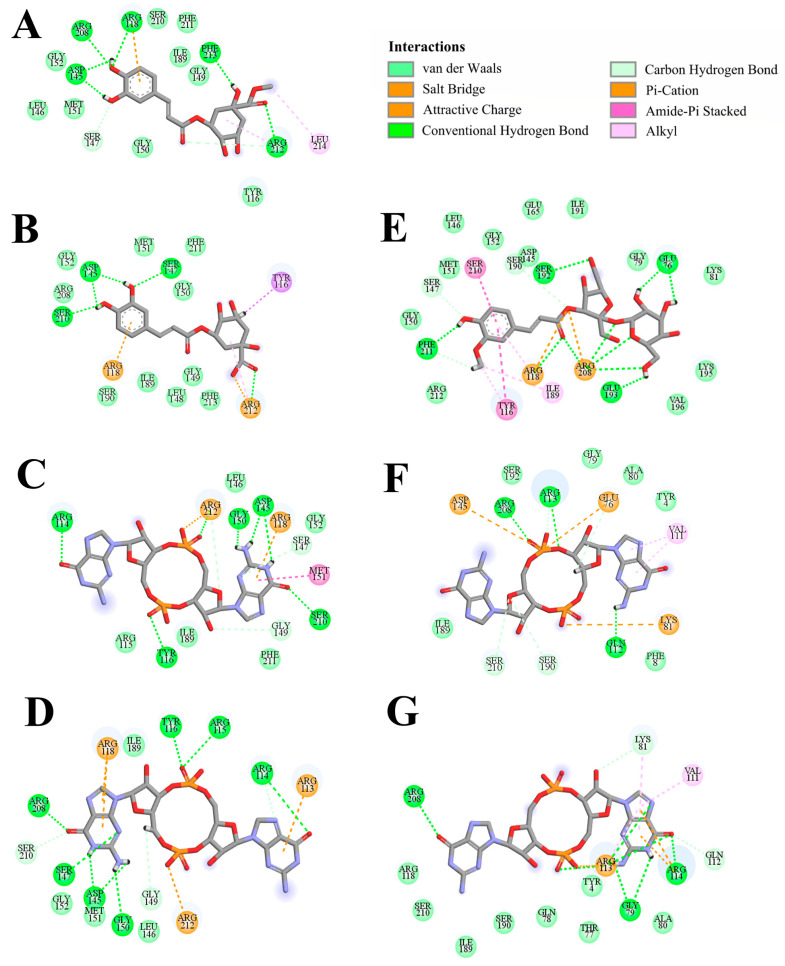
2D representations of the binding mode of YcgR with the docking ligands: (**A**) CAM; (**B**) CGA; (**C**) CDG-PilZ; (**D**) CDG-dual-PilZ; (**E**) Si-A5; (**F**) CDG-N domain; (**G**) CDG-dual-N domain. The ligands are shown in stick representation, with carbon atoms colored gray, oxygen atoms in red, nitrogen atoms in blue, and phosphorus atoms in orange.

Next, the 2D representations of the binding mode of ligands with YcgR are depicted in Figure 8. As can be seen, YcgR-CAM, YcgR-CGA, YcgR-CDG-PilZ, and YcgR-CDG-dual-PilZ displayed a similar binding mode, forming hydrogen bonds with residue Asp145. Interestingly, Arg212 was identified as a common key residue from the residue energy decomposition analysis, but it played various contributions for these four complexes. In detail, the hydrogen bond was observed between ARG212 and YcgR-CAM, YcgR-CGA, and YcgR-CDG-PilZ and attractive charges were found in ARG212 with YcgR-CDG-dual-PilZ. In addition, hydrogen bonds with residue ARG208 were observed in YcgR-Si-A5, YcgR-CDG-N domain, and YcgR-CDG-dual-N domain systems. Besides, van der Waals, carbon-hydrogen bond, Pi-cation, and Alkyl were also observed in the simulated systems. Additionally, the hydrogen bonds observed in CDG-bound systems (Arg113, Arg114, Arg115, Asp145, Ser147, and Arg208) were consistent with the previous study [10]. It is worth noting that Arg118 had favorable interactions with the benzene ring or hydroxyl group of caffeoylquinic acid compounds, while it had unfavorable interactions with the nitrogen atom of c-di-GMP by electrostatic repulsion. An in-depth analysis of Arg118 and the residues around it was performed. Conformation changes of Arg118 were induced by electrostatic interactions of Asp145, which is one of the key residues driving ligand binding with YcgR. Consistent with previous studies [9,10], the YcgR^Arg118Asp^ mutant is incapable of associating with c-di-GMP and interacting with the FliG protein.

## 3. Materials and Methods

### 3.1. Molecular Docking Study

The X-ray crystallographic structure of the YcgR protein in complex with c-di-GMP from *Escherichia coli* (PDB ID: 5Y6F [10]) was retrieved from the Protein Data Bank. Then, the structure was prepared using the Protein Preparation Wizard [28] in the Maestro interface of the Schrödinger 2023 suite, which involved adding hydrogen atoms, filling in the missing structures (residues 123–124, 156–160, and 195–203), and assigning charges and protonation states at pH 7.0. Subsequently, the structure was minimized using the OPLS-2005 force field [29]. The binding site for each compound was defined within a 10 Å × 10 Å × 10 Å cube centered on the mass center of the co-crystallized ligand, c-di-GMP, using the receptor grid generation tool in Glide, Schrödinger 2018. Sixty compounds previously isolated from *Pyrrosia lingua* were used for molecular docking. The 3D structures of these compounds were generated using the LigPrep module of Schrödinger 2018 based on their initial chirality. Protonation states and tautomers were assigned using Epik at a pH range of 7.0 ± 2.0, and up to 32 stereoisomers were allowed for each compound, with all other parameters set to default. In addition, the ligand efficiency (LE) for each compound based on the docking scores and the number of non-hydrogen atoms were also calculated based on the LE = (docking score)/(number of heavy atoms). According to good docking scores and key binding interactions, three compounds, including chlorogenic acid, sibiricose A5, and 3-*O*-caffeoylquinic acid methyl ester, were selected and purchased for further bioactivity validation.

### 3.2. In Vitro Antibacterial Assay

#### 3.2.1. Instruments and Chemicals

The following instruments were used: Haloes Caliper Circle of Inhibition Meter (Haloes Caliper, Shanghai, China), Hve-50 autoclave, and HCB-1300V medical ultra clean table (Haier, Qingdao, China). The following chemicals and materials were applied. Levofloxacin hydrochloride injection was purchased from CSPC OUYI Pharmaceutical Co., Ltd. (Taizhou, China), and chlorogenic acid, sibiricose A5, and 3-*O*-caffeoylquinic acid methyl ester were all purchased from Vicci Biotech Co., Ltd. (Chengdu, China). LB agar, powder, LB Broth, powder, 1% crystal violet ammonium oxalate solution, and phosphate buffer saline (PBS) were all purchased from Beijing Solepol Science and Technology Co., Ltd. (Beijing, China). Blank drug-sensitive paper tablets were purchased from Shanghai Qiyin Biotechnology Co., Ltd. (Shanghai, China). Ninety-six-well cell culture plates were purchased from Wuhan Seville Technology Co., Ltd. (Wuhan, China). UPEC (ATCC 10389) was provided by the laboratory of the College of Life Sciences, Guizhou University (Guiyang, China). Clinical isolates (*E. coli* 64222 and *E. coli* 55758) were provided by the Second Clinical Affiliated Hospital of Guizhou University of Traditional Chinese Medicine, and both were resistant to levofloxacin hydrochloride.

#### 3.2.2. Disk Diffusion Method

The concentrations of caffeoylquinic acid compounds, levofloxacin hydrochloride, and their combinations were set as 400 μg/mL, 200 μg/mL, 100 μg/mL, 50 μg/mL, and 25 μg/mL, respectively. The blank drug-sensitive paper (6 mm × 1 mm) was soaked in the above-mentioned compound solutions. Bacterial suspensions with a concentration of 1.5 × 10^8^ CFU/mL were evenly inoculated onto LB agar plates using sterile cotton swabs. The sterile water was used as the blank control. UPEC was placed in an incubator at 37 °C for 12 h. The experiments were repeated three times. Inhibition zone diameters (mm) were read by the Circle of Inhibition Measuring Instrument [30,31,32]. In addition, the MIC of levofloxacin hydrochloride in combination with caffeoylquinic acid compounds was determined using the same disk diffusion method mentioned above, which was used to evaluate the antibacterial activity of the combinations.

#### 3.2.3. Minimum Inhibitory Concentration

MIC is the lowest concentration of a drug or chemical that inhibits bacterial growth under in vitro conditions. The antimicrobial activities of each caffeoylquinic acid compound and antibiotic were determined by broth microdilution method with the 96-well plate [33,34]. UPEC was selected as the test organism, and incubated in a shaking incubator at 37 °C, 150 rpm for 12 h to revive the strain. LB Broth powder was used to culture bacteria. Serial two-fold dilutions for each caffeoylquinic acid compound and antibiotic were carried out ranging from 2000 μg/mL to 15.6 μg/mL and from 800 μg/mL to 6.25 μg/mL in a 96-well plate, respectively. A total of 100 µL bacterial suspension (1.5 × 10^8^ CFU/mL) and 2 μL TTC (0.5%) were added to each well. LB Broth, a powder containing UPEC, was set as a negative control. UPEC was cultured at 37 °C for 24 h. All the experiments were repeated three times.

#### 3.2.4. Microdilution Checkerboard Method

The combined effects of the caffeoylquinic acid compounds and the antibiotic were evaluated using the microdilution checkerboard method. Specifically, the activity of each combination was measured in a two-dimensional checkerboard fashion (8 × 8). The concentration changes were monitored by the MICs measured above. Each drug was used at seven increasing (two-fold) concentrations (1/32 × to 2  × MIC). A total of 100 μL bacterial suspension (1.5 × 10^8^ CFU/mL) was added. The microtiter plates were cultured at 37 °C for 24 h.

The fractional inhibitory concentration (FIC) index was calculated according to the formula as follows:

$$\mathrm{FIC}=\frac{{\mathrm{MIC}}_{\left(A\;\mathrm{in}\;\mathrm{the}\;\mathrm{presence}\;\mathrm{of}\;B\right)}}{{\mathrm{MIC}}_{\left(A\;\mathrm{alone}\right)}}+\frac{{\mathrm{MIC}}_{\left(B\;\mathrm{in}\;\mathrm{the}\;\mathrm{presence}\;\mathrm{of}\;A\right)}}{{\mathrm{MIC}}_{\left(B\;\mathrm{alone}\right)}}$$

The effects of the combinations of caffeoylquinic acid compounds and antibiotics were evaluated based on the FIC index: the FIC index ≤ 0.5 (Synergistic), 0.5 < FIC index ≤ 1 (additive), 1 < FIC index ≤ 2 (indifferent), FIC index > 2.0 (antagonistic), respectively [35,36].

#### 3.2.5. Crystal Violet Assay

The antibiofilm activity of each sample was determined using crystal violet assay in 96-well plates according to the method of Bai J et al. [37,38,39]. A total of 100 μL of the drug solutions at the concentrations of the MIC and 1/2 MIC were added to the 96-well plate, and then 100 μL of the bacterial suspension at a concentration of 1.5 × 10^8^ CFU/mL was added to each well. UPEC was incubated for 24 h, 36 h, 3 d, 5 d, and 7 d at 37 °C. All the experiments were performed three times. A total of 100 μL of LB Broth, powder and 100 μL of bacterial suspension were set as the negative control group. A total of 200 μL of LB Broth powder without the drugs was set as the blank control group. Each well was washed three times with PBS, and then fixed with methanol for 15 min. After drying, 1% crystal violet was added to each well and incubated for 15 min. Each well was washed with PBS. The plates were dried at 55 °C and 200 μL of 33% acetic acid was added. After 15 min, the absorbance at 590 nm was measured using an enzyme counter.

#### 3.2.6. Swimming Motility Assay

The swimming motility assay was performed according to Gao et al. with slight modifications [40]. In brief, a volume of 2.5 μL of the bacterial suspension (OD_600_ = 0.1) was inoculated into a swim plate containing (0.5% tryptone, 0.5% sodium chloride, and 0.3% agar), whose final drug concentration was set as 1/2 MIC. After 16 h of incubation at 37 °C, the diameter of the colonies occupied by each strain was measured. All experiments were conducted at least three times.

#### 3.2.7. Extraction and Quantification of c-di-GMP

C-di-GMP was extracted from UPEC (ATCC 10389) using the method previously described [41]. Following the cultivation of the bacteria during the late exponential growth phase, the cell suspension was diluted 50-fold in sterile LB broth containing the drugs at a concentration of half MIC. This mixture was then incubated at 37 °C with shaking at 200 rpm until the optical density at 600 nm (OD_600_) reached approximately 0.6 to 0.8, indicating mid-log phase growth. Two milliliters of planktonic culture were centrifuged at 10,000× *g* for 5 min at 4 °C. Bacterial cells were harvested and resuspended in 2 mL ice-cold PBS, incubated at 100 °C for 5 min, and sonicated for 15 min (power, 100%; frequency, 37 kHz) in an ice-water bath. After centrifugation, the supernatant containing c-di-GMP was collected, and the pellet was resuspended in 2 mL of ice-cold PBS and reextracted another two times. Intracellular c-di-GMP levels were quantified using a c-di-GMP ELISA kit (Mlbio, Shanghai, China). The total protein concentration in the supernatant was measured using a Pierce bicinchoninic acid (BCA) protein assay kit (Solarbio, Beijing, China). Intracellular c-di-GMP levels were reported as pmol/mg protein.

#### 3.2.8. qRT-PCR Analysis

UPEC was cultured with shaking at 37 °C until reaching the logarithmic phase (OD_600_ = 0.4) and subsequently incubated with drugs at a concentration of 1/4 MIC for 4 h. Total RNA was extracted using the M5 EASY spin plus kit (Mei5bio, Beijing, China). Subsequently, the RNA was reverse transcribed to cDNA using the M5 Sprint qPCR RT kit with gDNA remover (Mei5bio, Beijing, China). Real-time PCR was conducted using the Q2000C (LongGene instruments, Hangzhou, China), employing a mixture of primers and 2 × M5 HiPer SYBR Premix EsTaq (Mei5bio, Beijing, China). The 16S rRNA gene was utilized as the reference gene for the strain (Table 5). The Ct value for each sample was measured in triplicate, and the 2^−ΔΔCt^ method was employed to calculate the mRNA expression levels of YcgR.

### 3.3. Molecular Dynamics Simulations

Table 2 shows that three compounds, including chlorogenic acid, sibiricose A5, and 3-O-caffeoylquinic acid methyl ester, achieve significant synergistic effects with levofloxacin hydrochloride, as evidenced by their FIC values under 0.5. To further explore antibiofilm mechanisms, three docked structures (YcgR_CAM, YcgR_CGA, and YcgR_Si-A5) were derived from the molecular docking process detailed in the Section 3. Additionally, various configurations of the YcgR protein, including the unbound YcgR-apo, YcgR-CDG-dual with two c-di-GMP molecules (PDB ID: 5Y6F [10]), YcgR-CDG-PilZ with one c-di-GMP molecule near the PilZ domain, and YcgR-CDG-N-domain with one c-di-GMP molecule near the YcgR-N domain, were constructed. These configurations result in seven systems: YcgR-apo, YcgR-CDG-dual, YcgR-CDG-PilZ, YcgR-CDG-N domain, YcgR-CAM, YcgR-CGA, and YcgR-Si-A5.

Three independent 500-ns MD simulations for the seven systems were conducted using the AMBER 20 package [43]. The resp charges for ligands were generated utilizing Gaussian 16 combined with the Antechamber module in the AMBER 20 package. The protein YcgR and ligands were described by the ff14SB [44] and GAFF [45] force fields, respectively. Then, each system was neutralized with an appropriate amount of counterion (Cl^−^ or Na^+^), followed by solvation with TIP3P [46] water molecules in an orthogonal box, leaving at least 10 Å between the solute atoms and the box boundary. The cutoff distance for nonbonded interactions was set to 10 Å [47] In addition, the particle mesh Ewald (PME) [47] summation method was implemented to deal with Coulombic interactions, and the Shake [48] algorithm was applied to restrain bond vibrations involving hydrogen atoms. Four steps, including energy minimization, heating, equilibration, and production runs, were performed for the MD simulations. At first, four minimizations were carried out by employing the steepest descent and the conjugate gradient algorithm through 27,000 steps with the force constants of 5.0, 2.0, 0.1, and 0 kcal/mol/Å^2^ to all heavy atoms, protein backbone, and C_α_ atoms, respectively. Following the minimization steps, each system was heated from 0 K to 310 K with a force constant of 5.0 kcal/mol/Å^2^ for all heavy atoms. Then, all systems were equilibrated through 3000 steps with harmonic restraints of 1.0, 0.5, and 0.1 kcal/mol/Å^2^ on all heavy atoms, protein backbone, and C_α_ atoms, respectively. Next, a long 10-ns equilibration was conducted with a harmonic restraint of 0.1 kcal/mol/Å^2^. Finally, three independent 500-ns unrestrained MD simulations were simulated with randomized initial atomic velocities for each system.

### 3.4. Molecular Mechanics Generalized Born Surface Area Calculation

To evaluate the binding affinity of ligands on YcgR, the binding free energy and per-residue free energy decomposition were calculated using the MM/GBSA [49] method for each system. At first, water molecules and counter ions were extracted from the MD trajectory, and then 500 snapshots at time intervals of 25 ps were taken from the last 50-ns MD simulation. For each snapshot, the binding free energy ($\Delta G_{\mathrm{bind}}$) was calculated using the following formula:

$$\Delta G_{\mathrm{bind}}=\Delta G_{\mathrm{complex}}-\Delta G_{\mathrm{receptor}}-\Delta G_{\mathrm{ligand}}$$

where

$$\Delta G_{\mathrm{bind}}=\Delta E_{\mathrm{MM}}+\Delta G_{\mathrm{sol}}-T\;\Delta S$$

$\Delta E_{\mathrm{MM}}$ is the change in the molecular mechanics energy upon complexation in the gas phase, $\Delta G_{\mathrm{sol}}$ is the change in solvation free energy, and T ΔS is the change of conformational entropy associated with inhibitor binding. However, the entropy contribution is not considered here because our goal is not to obtain the absolute Gibbs energy. $\Delta G_{\mathrm{MM}/\mathrm{GBSA}}$ was averaged over the three parallel MD simulations, and per-residue free energy decomposition was determined by summing the energy components of each residue involved in the stabilization of the complex.

### 3.5. Statistical Analysis

In this study, all data were recorded as means ± standard deviations (SD), and a one-way analysis of variance (ANOVA) was conducted using SPSS Statistics version 26 software. *p* < 0.05 was considered significant. All experiments were performed at least in triplicate.

## 4. Conclusions

In summary, we illustrated the synergistic antibacterial and anti-biofilm mechanism of chlorogenic acid, sibiricose A5, and 3-*O*-caffeoylquinic acid methyl ester of *Pyrrosia lingua* combined with levofloxacin hydrochloride against UPEC. Our results showed that the combinations of the three compounds with levofloxacin hydrochloride enhanced the antibacterial activity against standard UPEC strain as the concentration increased. Moreover, the combined use led to MIC of levofloxacin hydrochloride and three caffeoylquinic acid compounds with a maximum reduction of 16-fold against the standard and clinically isolated UPEC strains. Chlorogenic acid and sibiricose A5 inhibited the biofilm formation of UPEC with levofloxacin hydrochloride at multiple time points. In addition, levofloxacin hydrochloride combined with chlorogenic acid or sibiricose A5 significantly increased the motility and c-di-GMP contents of UPEC, while the combined use of 3-*O*-caffeoylquinic acid methyl ester and levofloxacin hydrochloride had the opposite effects. The combination of levofloxacin hydrochloride and caffeoylquinic acid compounds exerted an inhibitory effect on the expression of the YcgR-1 gene. Two c-di-GMP within the binding site enhanced the stability of the complex, resulting in a more favorable and compact structure that promotes biofilm formation. Conversely, the presence of a single c-di-GMP molecule appeared to weaken this binding, thereby increasing protein’s motility. Notably, the interaction involving the c-di-GMP adjacent to the PilZ domain was more favorable compared to that near the YcgR-N domain. Additionally, the introduction of chlorogenic acid and sibiricose A5 was observed to decrease the motility of the YcgR protein, which in turn facilitated a more stable association with YcgR and was unfavorable for the association between c-di-GMP and YcgR. The van der Waals and direct electrostatic interaction were crucial driving forces for the binding between ligands and YcgR. These caffeoylquinic acid analogs have the potential to be used as antibiotic adjuvants for the treatment of urinary tract infections in the future. The study provides the foundation for solving the problem of antibiotic resistance and developing novel drugs for treating urinary tract infections.

## Figures and Tables

**Table 1:** Inhibition zone diameters for caffeoylquinic acid compounds and levofloxacin hydrochloride alone and in combination against *E. coli* ATCC 10389.

Concentration (μg/mL)	Inhibition Zone Diameters (mm) ^a^						
	CGA	Si-A5	CAM	LEV	LEV + CGA	LEV + Si-A5	LEV + CAM
25 ^b^	-	-	-	15.5 ± 0.1	17.1 ± 0.5 **	16.6 ± 0.2 **	16.4 ± 0.2 **
50	-	-	-	16.5 ± 0.4	19.3 ± 0.8 **	19.7 ± 0.4 **	17.0 ± 0.2
100	-	-	-	20.4 ± 0.5	23.6 ± 0.2 **	23.4 ± 0.2 **	24.7 ± 0.7 **
200	-	-	-	26.3 ± 0.3	27.7 ± 0.3 *	27.0 ± 0.3	27.8 ± 1.1 *
400	-	-	-	27.7 ± 0.2	29.3 ± 0.1	30.6 ± 1.8 **	29.6 ± 0.5 *
MIC ^c^	-	-	-	16.3 ± 0.4	16.4 ± 0.4	17.9 ± 0.7 *	19.2 ± 0.7 **

^a^ Values were the mean ± SD of three replicates. ^b^ Under the concentrations of LEV (25 μg/mL) and caffeoylquinic acid compounds (25 μg/mL). ^c^ Under the MIC concentrations of LEV (6.25 μg/mL) and caffeoylquinic acid compounds (250 μg/mL). */** The single (*) and double (**) asterisks represented *p* < 0.05 and *p* < 0.01, compared with LEV, respectively. - No activity.

**Table 2:** Fractional inhibitory concentration (FIC) index of antimicrobial combinations of caffeoylquinic acid compounds and levofloxacin hydrochloride against UPEC.

Strain	MIC (μg/mL)			FIC	MIC (μg/mL)		FIC	MIC (μg/mL)		FIC
	LEV	CGA	LEV + CGA		Si-A5	LEV + Si-A5		CAM	LEV + CAM	
*E. coli* ATCC 10389	6.25	250	0.78 + 62.50	0.38	250	0.78 + 31.25	0.25	250	0.78 + 31.25	0.25
*E. coli* 64222	31.25	≥1000	15.63 + 125	0.63	≥1000	3.91 + 125	0.38	1000	7.81 + 62.50	0.31
*E. coli* 55758	31.25	≥1000	15.63 + 62.50	0.56	≥1000	15.63 + 125	0.63	≥1000	15.63 + 250	0.75

**Table 3:** Components of the binding free energy between YcgR and three ligands averaged in the last 50 ns of the three replicates (kcal/mol).

Contribution	YcgR-CAM	YcgR-CGA	YcgR-Si-A5
Δ*E*_vdw_	−30.29 ± 2.69	−26.57 ± 0.63	−40.46 ± 2.57
Δ*E*_ele_	−67.49 ± 4.92	−217.26 ± 5.85	−110.06 ± 31.49
Δ*G*_pol,sol_	72.99 ± 3.73	216.30 ± 5.39	115.10 ± 27.97
Δ*G*_npol,sol_	−4.80 ± 0.34	−4.35 ± 0.08	−7.06 ± 0.36
Δ*E*_MM_	−97.77 ± 7.11	−243.82 ± 5.23	−150.52 ± 29.20
Δ*G*_sol_	68.19 ± 3.41	211.95 ± 5.44	108.04 ± 27.76
Δ*G*_MM/GBSA_	−29.59± 3.70	−31.87 ± 0.49	−42.48 ± 1.45

**Table 4:** Components of the binding free energy between YcgR and CDG averaged in the last 50 ns of the three replicates (kcal/mol).

Contribution	YcgR-CDG-PilZ	YcgR-CDG-N Domain	YcgR-CDG-dual-PilZ	YcgR-CDG-dual-N Domain
Δ*E*_vdw_	−55.25 ± 3.57	−43.13 ± 5.82	−58.16 ± 0.85	−44.58 ± 0.47
Δ*E*_ele_	−103.98 ± 36.09	−140.00 ± 28.44	−134.06 ± 1.04	−166.02 ± 7.96
Δ*G*_pol,sol_	121.10 ± 33.93	155.29 ± 29.87	143.18 ± 1.97	176.22 ± 5.26
Δ*G*_npol,sol_	−5.99 ± 0.41	−5.45 ± 0.58	−6.39 ± 0.03	−6.14 ± 0.03
Δ*E*_MM_	−159.23 ± 39.27	−183.13 ± 28.71	−192.67 ± 1.11	−210.60 ± 8.38
Δ*G*_sol_	115.11 ± 33.62	149.83 ± 29.48	136.79 ± 1.98	170.08 ± 5.24
Δ*G*_MM/GBSA_	−44.12 ± 5.97	−33.30 ± 1.50	−55.89 ± 1.04	−40.52 ± 3.20

**Table 5:** Sequences of primers used in this study.

Primer Name	Sequence (5′-3′)	Reference
16SrRNA	F:GGCTGAAAAGCTGCATTACC	[42]
	R:CATCAGGCCGATGTTACCTT	
YcgR-1	F:GCGCATTACTGGAAACAGC	This study
	R:TTCATTCTTGCCATCAATCACT	

## Data Availability

Data are available within the manuscript or the Supporting Materials.

## Supplementary Materials

The following supporting information can be downloaded at https://www.mdpi.com/article/10.3390/molecules29235679/s1, Figure S1: Residue contribution to the binding of ligands considering three replicates: (A) YcgR-CAM; (B) YcgR-CGA; (C) YcgR-CDG-PilZ; (D) YcgR-CDG-dual-PilZ; Figure S2: Residue contribution to the binding of ligands considering three replicates: (A) YcgR-Si-A5; (B) YcgR-CDG-N domain; (C) YcgR-CDG-dual-N domain; Figure S3: A cartoon representation of YcgR-CAM (green), YcgR-CGA (cyan), YcgR-CDG-PilZ (magenta), and YcgR-CDG-dual-PilZ (yellow). The ligands were highlighted by the black box; Figure S4: A cartoon representation of YcgR-Si-A5 (pink), YcgR-CDG-N domain (silver), and YcgR-CDG-dual-N domain (purple). The ligands were highlighted by the black box.
