# Supplementary material for: Synergistic Effects of Pyrrosia lingua Caffeoylquinic Acid Compounds with Levofloxacin Against Uropathogenic Escherichia coli: Insights from Molecular Dynamics Simulations, Antibiofilm, and Antimicrobial Assessments
Source: Molecules. 2024 Nov 30;29(23):5679. doi: 10.3390/molecules29235679 (PMC11643949; doi:10.3390/molecules29235679)
Supplement: Supplementary file 1 [file molecules-29-05679-s001.zip › molecules-3235764-supplementary.pdf]

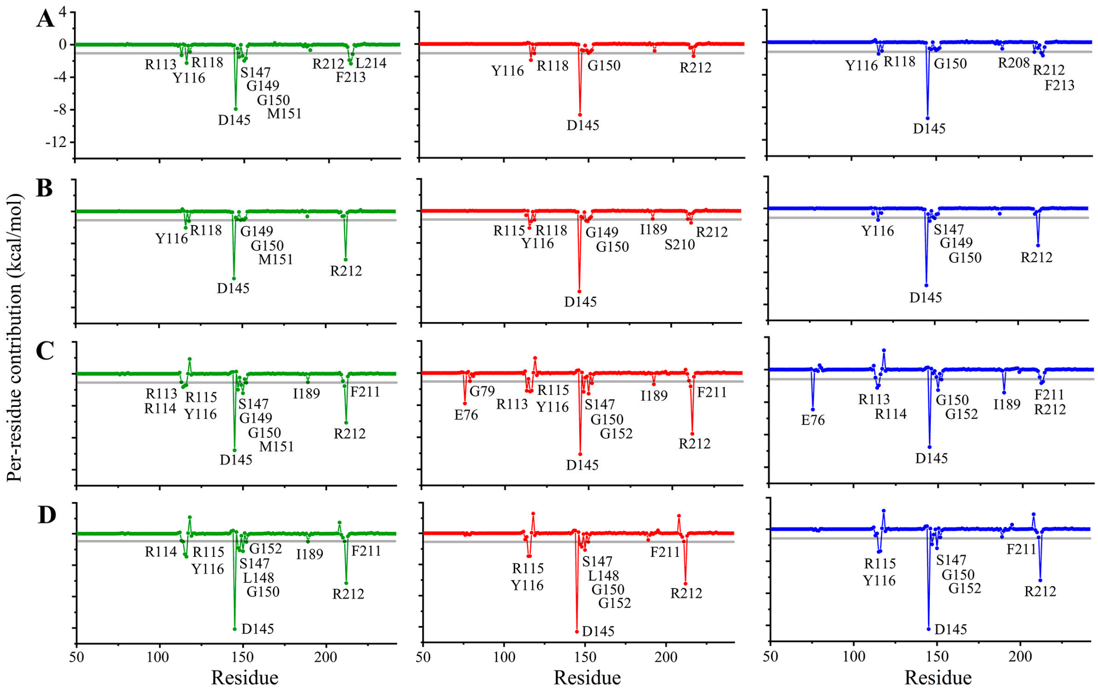

Figure S1. Residue contribution to the binding of ligands considering three replicates: (A) YcgR-CAM; (B) YcgR-CGA; (C) YcgR-CDG-PilZ; (D) YcgR-CDG-dual-PilZ.

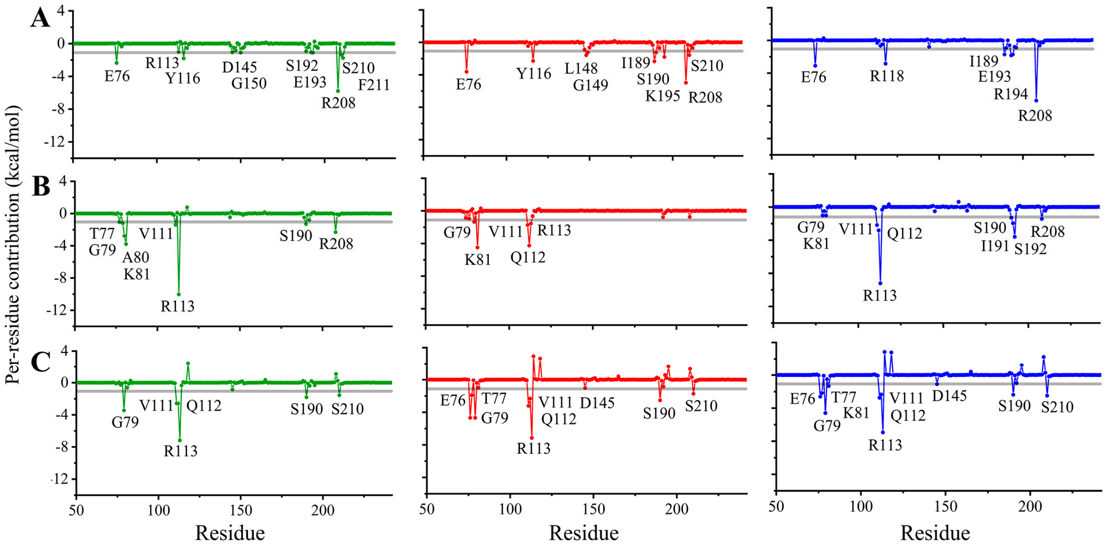

Figure S2. Residue contribution to the binding of ligands considering three replicates: (A) YcgR-Si-A5; (B) YcgR-CDG-N domain; (C) YcgR-CDG-dual-N domain.

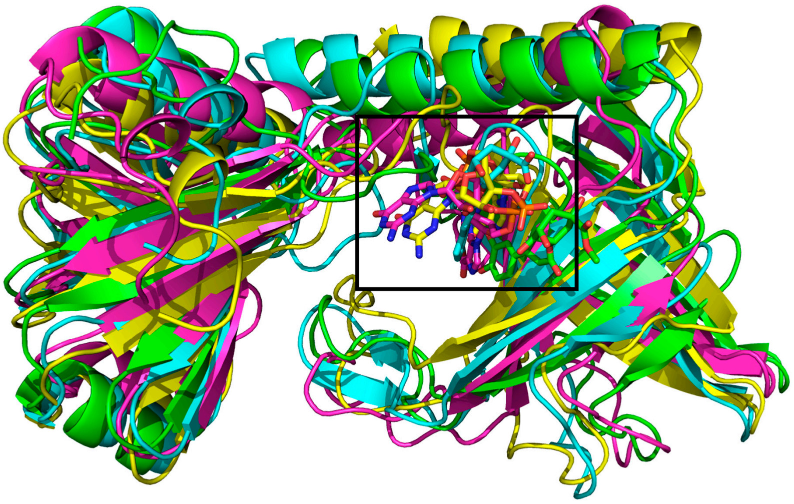

Figure S3. A cartoon representation of YcgR-CAM (green), YcgR-CGA (cyan), YcgR-CDG-PilZ (magenta), and YcgR-CDG-dual-PilZ (yellow). The ligands were highlighted by the black box.

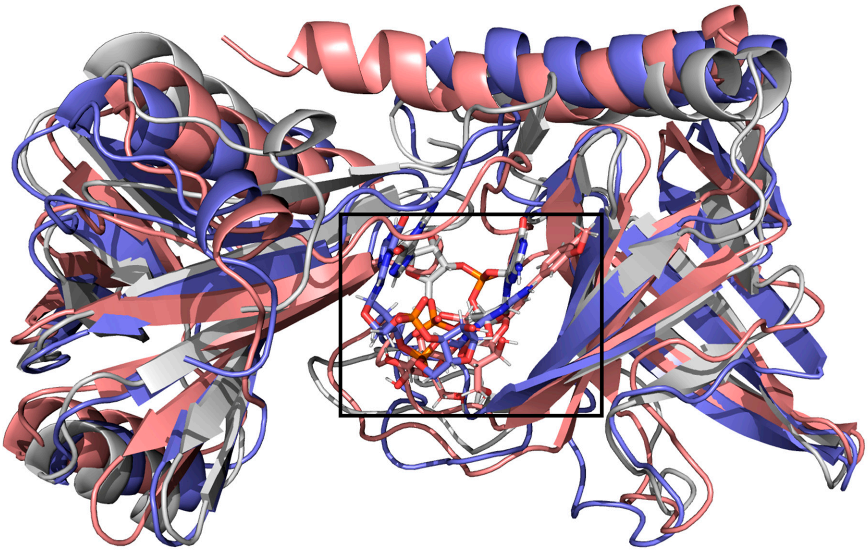

Figure S4. A cartoon representation of YcgR-Si-A5 (pink), YcgR-CDG-N domain (silver), and YcgR-CDG-dual-N domain (purple). The ligands were highlighted by the black box.
